# Gastric collision tumor: Case report of a rare adenocarcinoma and a typical carcinoid tumor

**DOI:** 10.3892/ol.2013.1313

**Published:** 2013-04-19

**Authors:** BETÜL ÜNAL, GÜLSÜM ÖZLEM ELPEK, TEKINALP GELEN, ALIHAN GÜRKAN, BÜLENT YILDIRIM

**Affiliations:** 1 Departments of Pathology, Akdeniz University Medical School, Antalya 07070, Turkey; 2 General Surgery, Akdeniz University Medical School, Antalya 07070, Turkey; 3 Internal Medicine, Akdeniz University Medical School, Antalya 07070, Turkey

**Keywords:** collision tumor, gastric adenocarcinoma, carcinoid

## Abstract

We report a case with features of gastric adenocarcinoma colliding with a typical carcinoid component. A 51-year-old female was admitted to the Department of Internal Medicine with complaints of epigastric pain. On physical examination of the patient there was significant epigastric tenderness and the CA19-9 level was higher than the normal titer value. An upper gastrointestinal endoscopy showed an ulcerated polypoid mass located on the cardiac region of the stomach. Pathological and immunohistochemical findings diagnosed as a collision tumor comprising both adenocarcinoma and carcinoid tumor. Metastasis of adenocarcinoma was found in 7 perigastric lymph nodes, while metastasis of the carcinoid was not detected. The admixture of neoplastic endocrine and nonendocrine cells, have been found infrequently in gastric tumors. The mixed tumors can be further classified into composite tumors that show an admixture of two histological components with histological transitions and collision tumors where the two components are not intermixed In general it is not easy to morphologically distinguish a collision tumor, from composite tumor. Microscopically, hematoxylin and eosin-stained tissue sections from two different areas of the mass revealed two different types of tumor; an intestinal type adenocarcinoma and a carcinoid tumor. We report a case with features of adenocarcinoma colliding with a typical carcinoid component, along with a review of the literature.

## Introduction

The admixture of neoplastic endocrine and non-endocrine cells have been identified infrequently in gastric tumors. For such lesions, four distinct categories are distinguished according to the tissue morphological features: carcinomas with interspersed endocrine cells, carcinoids with interspersed non-endocrine cells, mixed tumors and amphicrine tumors. Mixed tumors may be further classified into composite tumors, which exhibit an admixture of two histological components with histological transitions, and collision tumors, where the two components are not intermixed (1). While carcinoma with interspersed endocrine cells is the most frequent, the remaining tumors have rarely been reported in the stomach, with collision tumor being particularly unusual (1).

In the present study, we report a case with features of adenocarcinoma colliding with a typical carcinoid component, along with a review of the literature. The study was approved by the Ethics Committee of Akdeniz Unversity Medical Faculty, Antalya, Turkey. Written informed consent was obtained from the patient.

## Case report

A 51-year-old female was admitted to the Department of Internal Medicine of Akdeniz University Medical School, Antalya, Turkey, with complaints of epigastric pain. On physical examination there was significant epigastric tenderness. The biochemical analyses of blood and urine were within normal ranges. While the CEA level was not elevated, the CA19-9 level was higher than the normal titer value (82 U/ml; reference range, 0–40 U/ml). An upper gastrointestinal endoscopy showed an ulcerated polypoid mass located on the cardia. The histopathological examination of multiple biopsies from this lesion revealed a gastric adenocarcinoma. The patient underwent a total gastrectomy with lymph node dissection.

**Figure 1.**
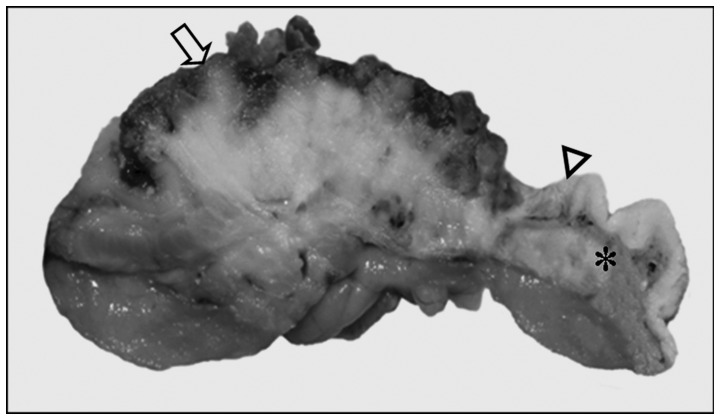
Macroscopic findings of the resected specimen. The two lesions were detected macroscopically; a large ulcerated polypoid mass (arrow) and a yellow lesion (asterisk) which was observed underneath the normal location of the gastric mucosa (arrowhead).

Macroscopic examination revealed an ulcerated polypoid mass measuring 2.5×1.5×2 cm. The dissected surface showed a yellow-white area surrounding the area of the ulcer. On closer inspection, a more yellow region (1 cm) was observed below the normal location of the gastric mucosal folds. This region and the ulcerated yellow area were abutted to each other (Fig. 1).

**Figure 2.**
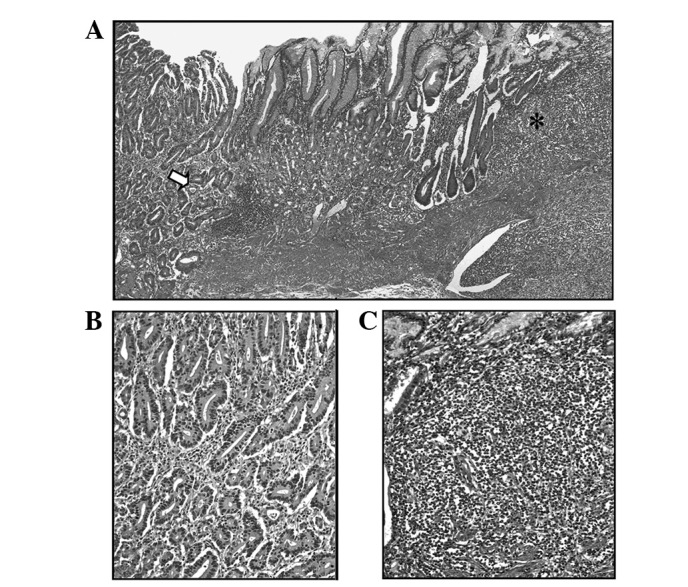
Histopathological findings of the collision tumor (hematoxylin and eosin staining). (A) Large polypoid tumor revealed moderately differentiated adenocarcinoma (arrow) and another tumor composed of small cells (asterisk). The two tumors were colliding in the gastric cardia and were clearly separated. Magnification, ×100. (B) Larger view of the adenocarcinoma forming gland-like structures (arrow in A). Magnification, ×200. (C) Larger view of the carcinoid tumor composed of a uniform population of small cells with solid patterns (asterisk in A). Magnification, ×200.

**Figure 3.**
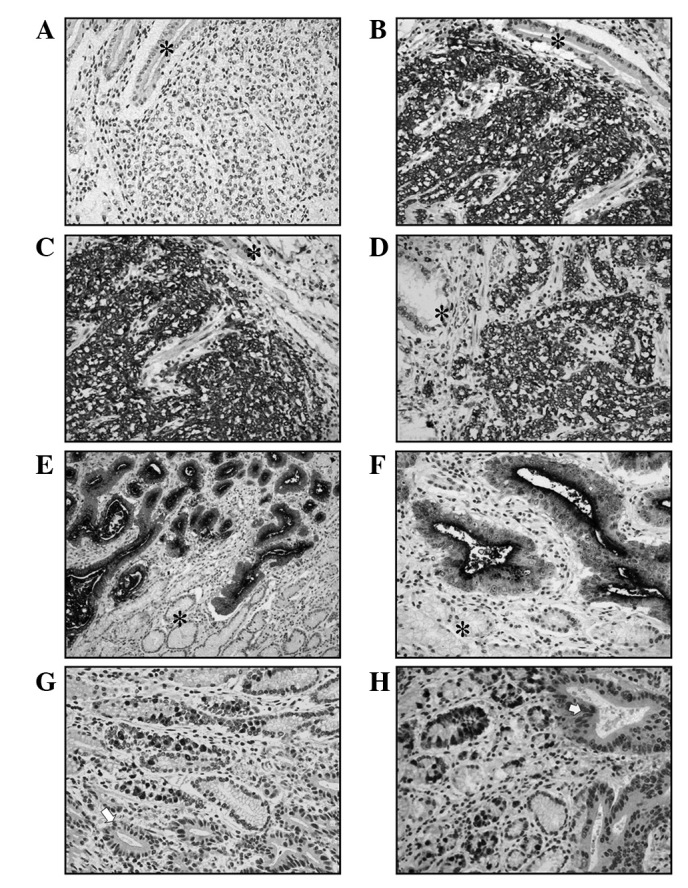
Immunohistochemical findings (avidin biotin peroxidase method counterstained with hematoxylin). (A) Tumor cells which formed the carcinoid component did not stain for CEA. (B–D) Cells markedly expressed NSE, chromogranin A and synaptophysin, respectively. The normal gastric mucosa is indicated by an asterisk. (E and F) Tumor cells of the adenocarcinoma stained for CEA, but not for (G) chromogranin A and (H) synaptophysin. Neuroendocrine cells of gastric glands stained for (G) chromogranin A and (H) synaptophysin. Arrows indicate tumor areas. (A–H) Magnification, ×400.

Microscopically, hematoxylin and eosin-stained tissue sections from two different areas of the mass showed two different types of tumor. One was a moderately differentiated intestinal type adenocarcinoma and the other was a tumor composed of a relatively uniform population of small cells with organoid, trabecular or focally solid patterns, suggesting neuroendocrine cell proliferation (Fig. 2). These cells exhibited a granular cytoplasm and an indistinct cytoplasmic border. Their nuclei had homogenous chromatin with indistinct nucleoli. Mitosis was infrequent (1/10 hpf) and necrosis was not observed. Immunohistochemical analysis showed that these cells had strong positivity for neuroendocrine markers (synaptophysin, chromogranin A and NSE) and this section of the tumor was diagnosed as a typical carcinoid tumor (Fig. 3). By contrast, adenocarcinoma cells expressed only CEA (Fig. 3). As the two tumors were distinctly separated from each other and no merging of tissue components was noted at the interface of the growth, the final diagnosis was a collision tumor composed of an adenocarcinoma and a carcinoid tumor of the stomach. The two tumors invaded the subserosal layer. While lymphatic permeation by the adenocarcinoma was noted, the carcinoid component was negative for lymphatic permeation. Vascular invasion was not observed for either component. Metastasis of the adenocarcinoma was identified in 7 perigastric lymph nodes among the 12 dissected lymph nodes, while metastasis of the carcinoid tumor was not detected.

### Clinical course

The patient succumbed to the tumor progression five months after surgery.

## Discussion

The present case involved a 51-year-old female with an ulcerated polypoid mass located at the cardiac region of the stomach. According to the pathological and immunohistochemical findings, the mass was diagnosed as a collision tumor comprising an adenocarcinoma and a carcinoid tumor. Although the presence of adenocarcinoma or carcinoid tumor individually is not notable, this collision tumor with two histopathological types in the stomach is only the eleventh case in the current literature. The patient in the present case report is younger than those reported in previous cases (mean, 61.7 years) and contrasts the frequent male predilection for this type of lesion (male/female, 4:1), demonstrating that collision tumors of this type are not only limited to older age groups or to males (Table I) (2–11).

As shown in the present case, gastric collision tumors comprising an adenocarcinoma and a carcinoid tumor are usually solitary lesions (2,4–11). The majority of previously reported tumors were located in the corpus (2,6,9–11). However, two previous cases were localized at the cardia (similar to the present case); one case was identified in the antrum and another in the fundus, suggesting that this tumor type may be encountered in different locations in the stomach (Table I) (3–5,7).

In general it is difficult to morphologically distinguish a collision tumor from a composite tumor. However, in the present case, the tumor comprised two components with different histopathological and immunohistochemical features. The two constituents were abutting each other without histological transition between them. Although metastasis of a composite tumor comprises both of the tissue components, metastasis of a collision tumor includes only a single tissue component (1). In our case report, lymph node metastases were monomorphic and had an adenocarcinoma component. Another problem in the correct diagnosis of collision tumors comprising an adenocarcinoma and a carcinoid tumor is that a diagnosis based on endoscopic biopsy may depend on the sampled site of the tumor. In these cases, if the biopsy specimen revealed only the carcinoid component, treatment and surgical intervention may be different.

Although pathogenic factors that contribute to the development of an adenocarcinoma or a carcinoid tumor alone have been extensively described, the pathogenesis of collision tumors comprising adenocarcinoma and carcinoid tumor is unclear. For instance, diet, genetics and infection with *Helicobacter pylori* may contribute to the development of gastric adenocarcinoma. Pernicious anemia and gastric atrophy may be a contributing factor for the development of a gastric carcinoid tumor (5). Alternatively, it has been postulated that carcinoid tumors are able to produce substances with a growth-promoting effect, which may account for the occurrence of a secondary tumor in the vicinity (12). In the present case, pernicious anemia and atrophic gastritis were not observed and the fasting serum gastrin value was not elevated. These tumors are considered to have arisen independently from multipotential epithelial stem cells and primitive neuroendocrine cells. In a previous report which carried out allelotyping analysis to study the genetic profiles of the endocrine and exocrine components of six mixed tumors of the gut, Furlan *et al* observed clonal divergence in a collision tumor, which was composed of a well-differentiated endocrine carcinoma associated with an adenocarcinoma (13). This finding confirms the existence of double tumors which grow next to each other coincidentally, yet exhibit different histogenesis and different tumorigenetic pathways.

The prognosis of this rare entity is unclear; however, from the few known cases it appears that the adenocarcinoma impacts more heavily on prognosis (1,5). The patient in this case succumbed to the tumor five months after gastric resection due to widespread liver metastasis of the adenocarcinoma.

In conclusion, the present case is the eleventh gastric collision tumor of its type, composed of an adenocarcinoma and a carcinoid tumor and this confirms their presence at this location. As further cases of this tumor type are reported, the clinicopathological properties and pathogenesis of this entity are likely to be revealed in more detail.

## Figures and Tables

**Table I.** Summary of previous studies on gastric collision tumor composed of an adenocarcinoma and a carcinoid tumor.

Authors (Ref.)	Age (years)	Gender	Location
Yamashina M *et al* (11)	50	Male	Corpus
Chodankar CM *et al* (10)	69	Female	Corpus
Morishita Y *et al* (9)	49	Male	Corpus
Corsi A *et al* (8)	72	Male	Unknown
Camuñas Mohinelo FA *et al* (7)	66	Male	Cardia
Olinici CD *et al* (6)	68	Male	Corpus
Morishita Y *et al* (5)	84	Female	Cardia
Jayaraman A *et al* (4)	48	Male	Antrum
Doggui MH *et al* (3)	55	Male	Fundus
Mróz A *et al* (2)	56	Male	Corpus
Present case	51	Female	Cardia
